# Global distribution and diversity of ovine-associated *Staphylococcus aureus*^☆^

**DOI:** 10.1016/j.meegid.2013.09.008

**Published:** 2014-03

**Authors:** Edward M. Smith, Polly F. Needs, Grace Manley, Laura E. Green

**Affiliations:** School of Life Sciences, University of Warwick, Gibbet Hill Road, Coventry CV4 7AL, UK

**Keywords:** *S. aureus*, Ovine, Caprine, MLST, *spa* typing, Global diversity

## Abstract

*Staphylococcus aureus* is an important pathogen of many species, including sheep, and impacts on both human and animal health, animal welfare, and farm productivity. Here we present the widest global diversity study of ovine-associated *S. aureus* to date. We analysed 97 *S. aureus* isolates from sheep and sheep products from the UK, Turkey, France, Norway, Australia, Canada and the USA using multilocus sequence typing (MLST) and *spa* typing. These were compared with 196 sheep isolates from Europe (*n* = 153), Africa (*n* = 28), South America (*n* = 14) and Australia (*n* = 1); 172 bovine, 68 caprine and 433 human *S. aureus* profiles. Overall there were 59 STs and 87 *spa* types in the 293 ovine isolates; in the 97 new ovine isolates there were 22 STs and 37 *spa* types, including three novel MLST alleles, four novel STs and eight novel *spa* types. Three main CCs (CC133, CC522 and CC700) were detected in sheep and these contained 61% of all isolates. Four *spa* types (t002, t1534, t2678 and t3576) contained 31% of all isolates and were associated with CC5, CC522, CC133 and CC522 respectively. *spa* types were consistent with MLST CCs, only one *spa* type (t1403) was present in multiple CCs. The three main ovine CCs have different but overlapping patterns of geographical dissemination that appear to match the location and timing of sheep domestication and selection for meat and wool production. CC133, CC522 and CC700 remained ovine-associated following the inclusion of additional host species. Ovine isolates clustered separately from human and bovine isolates and those from sheep cheeses, but closely with caprine isolates. As with cattle isolates, patterns of clonal diversification of sheep isolates differ from humans, indicative of their relatively recent host-jump.

## Highlights

• 97 global ovine *S. aureus* isolates characterised using MLST and *spa* typing.
• Majority of 261 global ovine isolates belong to one of three clonal complexes (CC).
• One CC has spread across the New World; two are restricted to Europe and Africa.
• Clonal complex spread matches the route and timing of sheep domestication.
• Patterns of clonal diversification of sheep isolates differ from human isolates.

## 1 Introduction

*Staphylococcus aureus* is widely recognised as a bacterial species that can colonise and infect a variety of hosts including humans, farmed and companion animals and exotic species (Cookson et al., 2007; Espinosa-Gongora et al., 2012; Porrero et al., 2012; Sasaki et al., 2012; Smith et al., 2005a). Multilocus sequence typing (MLST) has become the classical technique for analysis of bacterial population structure, and has been used extensively in the analysis of *S. aureus* populations from a variety of human and animal sources (Enright et al., 2000; Espinosa-Gongora et al., 2012; Sasaki et al., 2012; Smith et al., 2005b; Smyth et al., 2009). Animal isolates of *S. aureus* are commonly assigned to host-specific clonal complexes (CCs) including CC97 in cattle and CC133 in sheep (Smith et al., 2005b; Smyth et al., 2009). However, the discriminatory ability of MLST is low when compared with other techniques such as PFGE or *spa* typing. Whilst PFGE is laborious and can lack reproducibility, *spa* typing is based on sequence data (Harmsen et al., 2003; Shopsin et al., 1999) and is reproducible and comparable between studies. In addition, *spa* types associate with MLST CCs (Strommenger et al., 2006), hence *spa* typing is often used as an initial screen of study isolates (Eriksson et al., 2013; Porrero et al., 2012).

Notwithstanding the premise of host-specificity, the zoonotic transmission of *S. aureus* from livestock to humans has received a great deal of attention recently (Fitzgerald, 2012; Fluit, 2012; Lamamy et al., 2013; Pantosti, 2012; Price et al., 2012; Verkade and Kluytmans, 2013), and is being reported increasingly (Garcia-Alvarez et al., 2011; Petersen et al., 2013). Of particular concern is the zoonotic transmission of methicillin-resistant *S. aureus* (MRSA) that has occurred in Europe over the last decade, and the suggestion that some livestock associated strains might have the ability to colonise and infect humans (Garcia-Alvarez et al., 2011).

The ability of *S. aureus* to switch hosts has contributed to its ubiquity in human and veterinary medicine. Recently it has been estimated that individual *S. aureus* lineages switched from human to bovid hosts (cattle, sheep and goats) at different times. The earliest switch was approximately 5429 years ago and resulted in the bovine-associated CC151 and ovine-associated CC130 (Weinert et al., 2012). Additional lineages have arisen in both cattle and sheep since this initial host switch, the timing of which coincides with historical estimates of domestication events (Guinane et al., 2010; Weinert et al., 2012). Also two *S. aureus* host back-jumps are proposed; that is two lineages of *S. aureus* (ST59/966/754 and ST25 in CC151 and CC97 respectively) that switched from human to bovine hosts that have now independently switched back to human hosts (Weinert et al., 2012). It is therefore highly plausible that additional back-jumps from bovine and other species could occur in the future. This may be more likely in the developing world where there is more frequent contact between humans and their animals than in more developed regions. However, this may be influenced by animal numbers. In developed regions the time spent with individual animals may be less, but the pathogen load may be higher (because of higher animal numbers). Studies of farm personnel indicate that there is zoonotic transmission of *S. aureus*, because farm workers are often colonised by the same *S. aureus* MLST or *spa*-type as found in livestock (Cui et al., 2009; Spohr et al., 2011), although this is not always the case (Smith et al., 2005a).

In dairy and suckler ewes (ewes rearing lambs for meat production) *S. aureus* is a major cause of clinical mastitis, with a reported annual incidence rate of 0–6.6% (Arsenault et al., 2008). Both clinical and subclinical intramammary infections reduce farm profitability, and impact on ewe health and welfare, and lamb growth rates. Mastitis has been estimated to cost £8.40 per ewe in the UK (Conington et al., 2008). With an estimated national flock of 14.8 million breeding ewes (EBLEX, 2012), this results in a potential cost to the UK sheep industry in excess of £120 M/annum. Infections are treatable with antibiotics but the mammary gland rarely returns to full function, and infection will often result in the formation of intra-mammary abscesses. Asymptomatic carriage of *S. aureus* occurs in the nares, vagina and on skin, and these sites can act as potential reservoirs of infection (Mørk et al., 2012) making prevention of disease difficult. In addition, the related subspecies, *S. aureus* subsp. *anaerobius* causes Morel’s disease in sheep, a condition that leads to the formation of abscesses close to, or within, superficial lymph nodes (de la Fuente et al., 2011; Elbir et al., 2010).

Until recently, few studies focused on the analysis of ovine strains of *S. aureus*, and those that have, characterised isolates from relatively restricted geographical regions. This provides detailed information about the strains circulating within a region, but little information on the spread and diversity of global populations of *S. aureus* that colonise and infect sheep. The aim of the current study was to characterise the diversity of *S. aureus* in sheep and sheep cheese by examining 97 new isolates from the UK, Turkey, France, Norway, Australia, Canada and the USA, together with 196 existing ovine profiles from Africa, Australasia, Europe and S. America, to investigate the diversity and spread of global ovine isolates, and how they compare to strains from other hosts.

## 2 Methods

### 2.1 The ovine dataset, source of isolates

A total of 97 *S. aureus* isolates from sheep/ovine cheese were analysed in this study, this included 24 isolates from clinical mastitis, subclinical intra-mammary infections (IMI) and intra-mammary abscesses of sheep in England, 11 from cases of clinical mastitis in Australia, one from a severe case of clinical mastitis in Canada, 12 from cases of clinical mastitis, subclinical IMI, gangrenous mastitis, intra-mammary abscesses and carriage in France, 13 from cases of clinical mastitis, subclinical IMI and carriage in Norway (Mørk et al., 2012; 2007), three from subclinical IMI in the USA (Spanu et al., 2011) and 33 isolates from sheep milk cheeses in Turkey (Ertas et al., 2010), The strains used are described in Supplementary dataset 1.

Cultures from England were isolated as described previously (Smith et al., 2011), and confirmed as *S. aureus* by positive tube coagulase test result and *nuc* gene amplification (Brakstad et al., 1992). All isolates supplied from elsewhere were checked for purity and confirmed as *S. aureus* as above; where only DNA was provided, positive *nuc* gene amplification was used to verify the isolate was *S. aureus*.

### 2.2 DNA extraction and multi-locus sequence typing from the ovine dataset

DNA was extracted using the NucleoSpin Tissue Kit (Machery-Nagel GmbH & Co. KG, Düren, Germany). MLST and *spa* typing were performed as described previously (Harmsen et al., 2003; Shopsin et al., 1999; Smith et al., 2005a; 2005b). Briefly, for MLST, the primers of Enright et al. (2000) were used to amplify seven gene fragments (*arcC*, *aroE*, *glpF*, *gmk*, *pta*, *tpi* and *yqiL*), and raw PCR products were shipped to LGC genomics (Berlin, Germany) for purification and sequencing using forward and reverse primers. Sequence files were aligned and manually edited, with allele number and sequence types (STs) assigned using the *S. aureus* MLST website (http://saureus.mlst.net/, last accessed 27th March 2013). Novel allele trace files and allelic profiles of novel STs were sent to the database curator for allele or ST assignment and entry into the database. For *spa* typing, primers 1095F and 1517R (Harmsen et al., 2003; Shopsin et al., 1999) were used to amplify the polymorphic x region of the *spa* gene in all isolates. PCR products were sequenced as described above and sequence files manually aligned. *spa* types were assigned using DNAGear (AL-Tam et al., 2012), and novel types submitted to the Ridom SpaServer database (http://spa.ridom.de/submission.shtml, last accessed 7th August 2012) for *spa* type assignment.

### 2.3 Construction of the dataset of all *S. aureus* profiles

In addition to the MLST and *spa* profiles of the 97 *S. aureus* isolates characterised in the current study, further MLST and *spa* profiles of ovine isolates were obtained by searching the PubMed literature database (http://www.ncbi.nlm.nih.gov/pubmed, last accessed 28th March 2013) for articles describing characterised isolates using the terms ‘ovine’ or ‘sheep’, ‘aureus’ and ‘MLST’. An additional search of the *S. aureus* MLST database using the keywords ‘sheep’ and ‘ovine’ was also carried out. Where information on farm of origin was available, only one example of each ST and/or *spa* type per farm was included in the dataset to minimise sampling bias. This produced an ovine *S. aureus* dataset that was used in the analyses described below (Supplementary dataset 1). Data on geographical origin and isolation site were retained in the dataset.

Example MLST and *spa* profiles of *S. aureus* strains from goats, cattle and humans formed a second dataset for comparison with the ovine dataset. Bovine and caprine profiles were obtained from the references identified in the ovine search, and human *S. aureus* profiles were obtained from descriptions of the analysis of large culture collections (Cookson et al., 2007; Feil et al., 2003). A full list of the isolates compared is presented in Supplementary dataset 2.

### 2.4 Data analysis

#### 2.4.1 Population diversity

Simpson’s indexes of diversity [*D*] were calculated for individual loci, ST and *spa* types using V-DICE (VNTR DIversity and Confidence Extractor; http://www.hpa-bioinformatics.org.uk/cgi-bin/DICI/DICI.pl, last accessed 1st August 2013). The ovine dataset was divided into clonal complexes using the conservative definition of six out of seven matching alleles using eBURST v3 (http://eburst.mlst.net/, last accessed 27th March 2013). Clonal complexes were named on the basis of the predicted ancestral strain, if there was no predicted ancestral strain they were classed as minor groups and were numbered arbitrarily. The global optimal eBURST (goeBURST) algorithm (Francisco et al., 2009), implemented in PHYLOViZ (Francisco et al., 2012) was used to visualise CCs, including both single and double locus variants, and was supplemented with isolate metadata including geographical origin and isolation site.

#### 2.4.2 Clonal diversification

Estimates of the rate of recombination during clonal diversification were made as described previously (Feil et al., 2003; Smith et al., 2005b). Briefly, single locus variant (SLV) STs were compared to their ancestral ST, variant alleles were identified and compared to determine nucleotide and amino acid changes. Alleles differing at a single nucleotide site that were unique to the ST within the ovine dataset were classed as having arisen by mutation; alleles not satisfying these criteria were considered to have arisen by recombination.

## 3 Results

### 3.1 Descriptive results

All 97 ovine-associated *S. aureus* isolates were successfully typed by MLST and *spa* typing. Twenty-two STs were detected, including three new alleles (*gmk_*191, *pta_*270, *tpi_*272) and four novel STs (2488, 2489, 2490, 2491); 37 *spa* types were identified including eight new types (t12378, t12379, t12380, t12381, t12382, t12383, t12384 and t12663). An additional 169 ovine-associated *S. aureus* strains were identified from the literature and 27 from the *S. aureus* MLST database. This generated an ovine dataset of 293 isolates for analysis containing profiles from Africa, Australasia, Eurasia (Turkey), Europe, N. America and S. America. There was a large proportion of European isolates, so these were separated into S. Europe (Italy, Spain), C. Europe (France, Germany), N. Europe (Denmark, Iceland, Norway, Sweden) and the UK & Ireland. The strains were isolated from sheep milk, nasal and vaginal carriage, intra-mammary abscesses, sheep cheeses and lymph node abscesses (*S. aureus* subsp. *anaerobious*; *n* = 24; Supplementary dataset 1).

### 3.2 Identification of clonal complexes

**Fig. 1:**
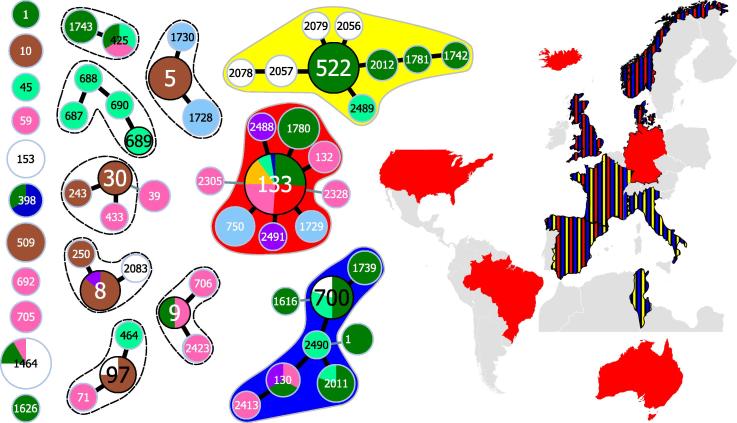
Origin of ovine-associated *S. aureus* isolates within clonal complexes, minor group and singleton STs. Numbers indicate ST, and circle size is proportional to the numbers of isolates. Putative ancestral STs have a black border, single locus variants are connected by thick black lines and double locus variants by narrow grey lines. Each circle is a pie chart, and colours within circles indicate the geographic location each ST was isolated from: UK and Ireland (purple), S. Europe (dark green), C. Europe (light green), N. Europe (pink), N. America (dark blue), S. America (light blue), Eurasia (brown), Australasia (orange) and Africa (white). The extent of the three main ovine-associated CCs is indicated by shading in red [CC133], blue [CC700/130] and yellow [CC522], and their geographic origins are indicated on the map in the same colours. The extent of the remaining CCs and minor group is indicated by a dashed line.

A total of 59 STs were identified in the 293 profiles, generating a Simpson’s index of diversity (*D*) of 0.86 (95% CI: 0.83–0.90). The 59 STs were divided into nine clonal complexes (CCs), one minor group and 16 singletons by eBURST. The singletons comprised 17.1% (*n* = 50) of all isolates; almost half of these (*n* = 24) were *S. aureus* subsp. *anaerobious*, the remainder consisted of milk, cheese and carriage isolates. Of the remaining 243 isolates, 180 were present in three clonal complexes: CC133 (*n* = 110), CC522 (*n* = 46) and CC700 (*n* = 24) (Supplementary dataset 1), the inclusion of double locus variants added two isolates to CC133 and three isolates to CC700 (Fig. 1). None of the other CCs or the minor group contained more than 15 isolates. CC133 contained isolates from Europe (all regions, *n* = 88), Australasia (*n* = 12), S. America (*n* = 8) and N. America (*n* = 2); CC522 contained isolates from S. Europe (*n* = 40), Africa (*n* = 5) and C. Europe (*n* = 1); CC700 contained isolates from Europe (all regions, *n* = 22) and Africa (*n* = 2). Isolates from all sites were present in the main three CCs, although there was only one sheep cheese isolate present in these CCs. The remaining 32 cheese isolates were distributed around four of the smaller CCs and singleton STs. There was no association between CC and sample type (milk, carriage, cheese or abscess). Five of the nine ancestral STs (55.5%) were isolated from more than one site whereas 46 / 50 (92.0%) derived or singleton STs were isolated from a single site. However of the 59 STs, 33 are represented in the dataset by a single isolate. On average there are more ancestral ST representatives (mean = 18.1, range 1–90) than derived/singleton STs (mean = 2.3, range 1–24).

### 3.3 *spa* type diversity

There were 219 isolates with 87 *spa* types in the ovine dataset, 51 *spa* types were present in a single isolate, and of the remaining 36 types, nine were found in five or more isolates (Supplementary dataset 1). Four *spa* types were present in ten or more isolates, three (t2678, t1534, t3576) were found in more than one sample type (milk, carriage, abscess), and one (t002) was only detected in sheep cheese; these *spa* types were associated with CC133, CC522, CC522 and CC5 respectively. *spa* type t1403 [*n* = 3] was the only type to be present in more than one CC (CC133, CC700 and a singleton). Eight *spa* types were detected in sheep cheeses; none of these were present in other sample types.

### 3.4 Comparison with other host species

**Fig. 2:**
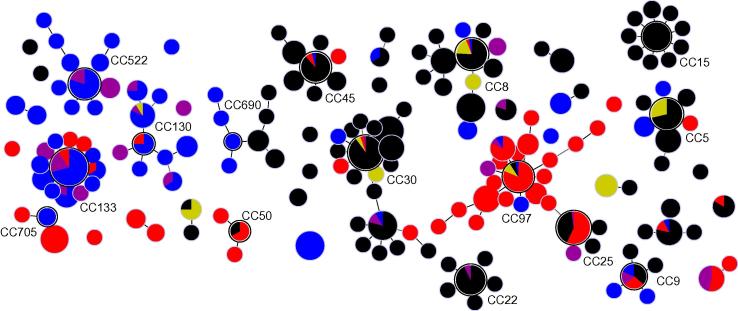
Host-association within *S. aureus* clonal complexes, minor groups and singleton STs. Each circle is a pie chart, and the size proportional to the numbers of isolates; colours indicate the host the ST was isolated from: sheep (blue), goats (purple), cattle (red), humans (black) and sheep cheeses (yellow). Putative ancestral STs identified by eBURST have a white border and the clonal complex number is given.

When compared to 172 bovine, 68 caprine and 433 human isolates, the 293 ovine isolates clustered separately from human and cattle strains; and the majority of caprine isolates were distributed between ovine- and bovine-associated CCs (Fig. 2). These 966 isolates represented 177 STs and 127 *spa* types (not all isolates were *spa* typed), and were split into 15 CCs, 10 minor groups and 28 singleton STs by eBURST. Our analysis of ovine isolates identified ST700 was the most likely ancestor of a clonal complex, and ST2490 was also placed centrally in the CC (Fig. 1). However analysis of the full multi-host dataset predicted ST130 as the ancestor, agreeing with previous studies of ovine-associated *S. aureus*. This variation in the identification of putative ancestral STs is due to initial analysis of a restricted dataset, and the assumptions contained within the analysis algorithm. Factors that are used to identify the putative ancestor include the number of variants the ST defines and the total numbers of isolates of a ST (Francisco et al., 2009). In the initial analysis, ST700 links to three other STs and is represented by multiple isolates; ST2490 links to four STs, however, it is only represented by a single isolate, so ST700 is the assumed ancestor. When a larger dataset is analysed, ST130 is identified as the putative ancestor of the CC because it has links to four STs (more than any other ST in the CC) and is represented by multiple isolates.

Using a threshold value of 50% of isolates within a CC or minor group to indicate host tropism, there are nine human-associated, seven bovine-associated and four ovine-associated clusters. All host species are present in CC9, with none dominating (43% human, 29% ovine, 14% bovine and 14% caprine), and Groups II and VI contain one bovine and one human isolate. GroupIV (*n* = 7) and GroupVII (*n* = 5) are associated with sheep cheese, these latter two groups contain two STs each (ST207/509 and ST10/145 respectively). All four of these STs have previously been detected in humans (Cookson et al., 2007; Feil et al., 2003; *S. aureus* MLST database [http://saureus.mlst.net/] last accessed 26th March 2013) so it is possible that these two groups are really human-associated, with humans contaminating cheese, but because they contained a greater number of cheese than human isolates in the current study, they are classed as cheese-associated.

Twenty *spa* types present in the ovine dataset were also present in cattle, goat or human populations. Four *spa* types detected in humans were found in sheep cheese (t002, t021), carriage (t044) and milk isolates (t015), and a third *spa* type detected in sheep cheese (t008) has previously been found in goat and human studies (Cookson et al., 2007; Porrero et al., 2012). The remaining 15 *spa* types present in both datasets were detected in cattle and/or goats, sheep milk, carriage and abscess samples.

The bovine isolates originated from the UK and Ireland, N. Europe, S. Europe, N. America and S. America, and so have less diverse geographical origin than the ovine strains. However they have a comparable, if marginally greater, core genome diversity (Table 1). Individual locus diversity of strains colonising the two hosts varies; the most variable locus in ovine-associated strains (*aroE*, Simpson’s *D* = 0.81) is amongst the least variable in bovine strains (Simpson’s *D* = 0.66) (Table 1).

### 3.5 Clonal diversification of ovine isolates

There were 19 SLVs in the three main ovine CCs, resulting in non-synonymous amino acid changes in 14 of the derived alleles (Table 2). Thirteen SLVs differed at one nucleotide site from their putative ancestor, and 11 of these were unique to their ST (Table 2). Of the two alleles that differed at a single site that were not unique to their ST: *tpi_*233 in ST2057 and *pta_*189 in ST2012; the former was only present in CC522 whereas *pta_*189 was in STs assigned to both CC522 [ST2012] and CC700 (specifically, ST1758 a DLV of ST2490). The remaining six SLVs differed from their putative ancestor at more than one nucleotide site, and with the exception of *pta_*188 in ST1742, all were present in other ovine-associated STs. Two loci (*gmk* and *yqiL*) change once during clonal diversification (both in CC522), however, their overall diversity varies greatly; *gmk* is the least diverse locus in ovine-associated strains, and *yqiL* is one of the most diverse. In contrast, *pta* changes six times but is one of the least diverse loci overall.

## 4 Discussion

We have characterised 97 new ovine *S. aureus* isolates with widespread geographical origins using MLST and *spa* typing, and combined them with 196 existing profiles to provide the largest study of the global distribution and diversity of ovine *S. aureus* to date. Previous studies, with a more limited geographical distribution of isolates, have associated CC130, CC133 and CC522 with small ruminants (de Almeida et al., 2011; Eriksson et al., 2013; Guinane et al., 2010; Porrero et al., 2012; Smyth et al., 2009), and these are confirmed in the broader study presented in the current paper. The key results from the current study are that the evolution of ovine *S. aureus* fits the geographic and temporal pattern of sheep domestication; that ovine and caprine isolates are similar, and distinct from human and cattle isolates; and that recombination is more common in livestock-associated strains of *S. aureus* than human-associated strains which appear more genetically stable.

CC700/130 contains ovine strains that were isolated from Europe and Africa. This lineage is estimated to have switched to bovid hosts approximately 5429 (range 3082–8981) years ago (Weinert et al., 2012), coinciding with the expansion of livestock domestication throughout the Old World. Our data indicate that this complex has not spread beyond this region, and whilst future studies might prove otherwise, the current data suggest that sheep selection (e.g. for meat) has influenced the existing phylogeography of ovine *S. aureus*. CC133, one of the three common CCs, has achieved widespread geographic distribution across the relatively developed regions of Europe, Australasia and the Americas. It is notable that there were no African isolates in CC133, and the presence of CC133 in Asian ovine *S. aureus* populations remains to be determined. The absence of CC133 in Africa might be explained by the timing and location of host switching, a recent estimate suggests the switch of CC133 from human to bovid hosts occurred approximately 3113 (range 1183–6113) years ago (Weinert et al., 2012). This follows the initial livestock domestication events of 8000–10,000 years ago (Bruford et al., 2003), and was probably during the period of specialisation for secondary products, such as wool, that occurred in Europe in the fourth millennium before present (B.P.) (Chessa et al., 2009). The lineage might then have spread and evolved with sheep populations across Europe and have been exported to the Americas and Australasia.

As with CC700/130, CC522 also appears to be restricted to Africa and Europe (almost exclusively S. Europe) (Gharsa et al., 2012; Porrero et al., 2012). There are currently no estimates of when this lineage switched hosts, and a calculation of timing is outside the scope of this study. However, based on the geographical distribution of strains, a host-switching event prior to secondary selection i.e. >4000–5000 years B.P. appears plausible. Collectively these data suggest that as sheep were domesticated and spread out from the domestication centre (southwest Asia) (Bruford et al., 2003), that *S. aureus* lineages were domesticated and spread with them; and that later periods of selection also selected for new small-ruminant associated *S. aureus* lineages that were then exported and spread with (and by) their hosts.

The detection of novel *spa* types is probably due to limited previous studies of ovine isolates from broad geographical origins. There is a greater diversity, and therefore discriminatory ability, of *spa* compared with MLST typing (Eriksson et al., 2013; Porrero et al., 2012), and there was a close relationship between *spa* types and MLST CCs; only one *spa* type was present in more than one CC. However *spa* typing did permit the discrimination of isolates with identical STs. Isolates from Australia (all ST133) were split into four *spa* types; two were novel, one (t3042) has been detected in Danish sheep populations (Eriksson et al., 2013), and one (t998) was detected in France (unknown host: http://spa.ridom.de/spa-t998.shtml, last accessed 7th August 2012). This adds to the evidence of a direct connection between European and Australian ovine isolates, possibly because of the introduction of sheep from Europe into Australia. Three isolates collected from a single sheep at two time-points [37_008, 37_010, 37_125; Supplementary dataset 1] were included in our study; *spa* typing reveals that two closely-related strains were present during this infection.

Ovine and caprine isolates are close relatives (Ben Zakour et al., 2008) and are adapting to, and diversifying within, their host(s) following evolutionarily recent host jumps (Guinane et al., 2010; Weinert et al., 2012). This close relationship might be because in many countries, such as Greece, sheep and goats are farmed together. They have different foraging habits, so rearing the two together has allowed traditional small scale farmers to take advantage of all available forage. Sheep milk can be combined with [up to 35%] goat milk to produce feta cheese (T. Papadopoulos, personal communication).

Differences in levels of diversity at individual loci between ovine and bovine isolates are indicative of host-specific adaptation. It is probable that these changes, or those linked to MLST alleles, contribute to the varying genome content between ovine, bovine and human isolates (Guinane et al., 2010; Resch et al., 2013), and improve the ability of the resultant strains to colonise/infect sheep and cattle, however, not all *S. aureus* lineages are host-specific. ST398 has rarely been detected in sheep (Eriksson et al., 2013; Viana et al., 2010) and was thought to be a contaminant in one study (Eriksson et al., 2013). However detection of the porcine-associated strain ST398 t034 (Overesch et al., 2011; Verhegghe et al., 2013) in independent isolates from the USA (Spanu et al., 2011) and Canada suggests that this strain is capable of adapting to, and infecting, new hosts. Elsewhere ST398 has been associated with a range of other host species (reviewed in Verkade and Kluytmans, 2013). Methicillin-resistant *S. aureus* (MRSA) ST398 is a rapidly emerging cause of human infection, often associated with livestock exposure. The first recorded cases of human infection with MRSA ST398 were approximately 10 years ago in central Europe (Voss et al., 2005); and in the Netherlands MRSA ST398 now accounts for up to 25% of MRSA cases in some regions (van Cleef et al., 2011). The jump of ST398 from humans to livestock was associated with the loss of some human virulence genes, but also the acquisition of tetracycline and methicillin resistance (Price et al., 2012). Thus the zoonotic and antibiotic resistance potential of this lineage, coupled with its spread to new hosts and geographical regions are causes for concern.

CC9 contains isolates from all hosts studied (Eriksson et al., 2013; Feil et al., 2003; Porrero et al., 2012; Smith et al., 2005b), and has also been associated with porcine and avian isolates (Armand-Lefevre et al., 2005; Fessler et al., 2011). This broad host range suggests this is a potentially zoonotic lineage. A recent report of CC9 bloodstream infections in humans not associated with livestock (Lamamy et al., 2013), might indicate that isolates within CC9 are becoming host-adapted and spreading within a host population. Similarly, strains from CC5 are being isolated from an increasing diversity of host species. It was traditionally considered a human-associated lineage (Cookson et al., 2007; Feil et al., 2003), but STs from CC5 have recently been reported in poultry (Smyth et al., 2009), sheep (de Almeida et al., 2011) and cattle (Aires-de-Sousa et al., 2007; Hata et al., 2010). STs from CC5 were also present in sheep cheeses in the present study. These isolates originated from a single source (Ertas et al., 2010), and few clustered with other strains isolated directly from sheep or sheep milk. This indicates that either the cheeses were contaminated with *S. aureus* during processing or that sheep in Turkey are colonised with STs more commonly associated with human hosts. The detection of apparently human-associated *spa* types within these isolates supports the contamination hypothesis, however, more detailed analysis of sheep milk and the resultant cheeses is required to determine the source of these strains.

The results of the current work together with results from cattle (Smith et al., 2005b) confirm that livestock CCs diversify by recombination more frequently than human CCs. Analysis of human isolates has suggested that strains are at least 15-fold more likely to diversify by point mutation than recombination (Feil et al., 2003); whereas ovine isolates are only 1.4 times more likely to diversify by mutation (11 out of 19 SLVs arose by mutation). The presence of alleles in more than one ST and CC indicates that alleles are being maintained within the population, and the high proportion of non-synonymous changes suggests that they result in functional changes. This altered population structure of livestock-associated *S. aureus* compared with human-associated *S. aureus* is likely due to *S. aureus* host adaptation. In contrast, human-associated strains are more genetically stable because they have been present in their host for a longer time.

## 5 Conclusions

There is considerable heterogeneity within global ovine *S. aureus* isolates. The majority of global ovine isolates typed to date belong to three main CCs; one CC has achieved widespread distribution across Europe and the New World and the remaining two appear confined to Europe and Africa. Ovine isolates are distinct from those that colonise and infect humans and cattle; and patterns of *S. aureus* clonal diversification in livestock differ from human isolates as the populations adapt to their (new) hosts.

## Figures and Tables

**Table 1:** *spa*, ST and MLST individual locus Simpson’s index of diversity (*D*) for ovine and bovine isolates.

Locus	Ovine *D* (95% CI^a^)	Bovine *D* (95% CI^a^)
*spa*	0.96 (0.95–0.97)	0.90 (0.87–0.93)^b^
ST	0.86 (0.83–0.90)	0.92 (0.90–0.93)
*arcC*	0.74 (0.69–0.78)	0.73 (0.69–0.77)
*aroE*	0.80 (0.71–0.84)	0.66 (0.59–0.74)
*glpF*	0.75 (0.72–0.79)	0.68 (0.62–0.74)
*gmk*	0.46 (0.39–0.53)	0.69 (0.62–0.75)
*pta*	0.55 (0.48–0.62)	0.70 (0.64–0.76)
*tpi*	0.78 (0.73–0.82)	0.59 (0.51–0.67)
*yqiL*	0.79 (0.71–0.82)	0.80 (0.76–0.84)

a 95% Confidence intervals.

b *spa* type data was only available for 16 bovine isolates.

**Table 2:** Single locus variants within CC133, CC700 and CC522.

CC^a^	Ancestral ST^b^	SLV^c^ ST^b^	Variant locus	Ancestral allele	SLV^c^ allele	No. nuc.^d^ differences	Amino acid change	Present in another ST^b^
**133**	133	2488	*pta*	7	270	1	Synonymous	No
	133	1780	*aroE*	66	95	10	Non-synonymous	Yes
	133	132	*aroE*	66	67	1	Non-synonymous	No
	133	1729	*pta*	7	182	1	Non-synonymous	No
	133	2491	*tpi*	50	272	1	Non-synonymous	No
	133	750	*arcC*	6	89	1	Synonymous	No
**700**	700	1739	*arcC*	6	194	1	Non-synonymous	No
	700	2490	*tpi*	95	14	6	Non-synonymous	Yes
	2490	2011	*arcC*	6	200	1	Synonymous	No
	2490	130	*tpi*	14	58	5	Non-synonymous	Yes
	130	2413	*glpF*	45	294	1	Synonymous	No
**522**	522	2079	*pta*	7	218	1	Synonymous	No
	522	2056	*glpF*	45	272	1	Non-synonymous	No
	522	2489	*gmk*	2	191	1	Non-synonymous	No
	522	2057	*tpi*	15	233	1	Non-synonymous	Yes
	2057	2078	*pta*	7	4	2	Non-synonymous	Yes
	522	2012	*pta*	7	189	1	Non-synonymous	Yes
	2012	1781	*yqiL*	5	4	6	Non-synonymous	Yes
	1781	1742	*pta*	189	188	2	Non-synonymous	No

a Clonal complex.

b Sequence type.

c Single locus variant.

d Nucleotide.

## Appendix A Supplementary data

Supplementary dataset 1 This document contains supplementary information.

Supplementary dataset 2 This document contains supplementary information.
